# Problems and Opportunities in the use of Bioelectrical Impedance Analysis for Assessing Body Composition During Ketogenic Diets: A Scoping Review

**DOI:** 10.1007/s13679-024-00573-0

**Published:** 2024-05-27

**Authors:** Antonio Paoli, Francesco Campa

**Affiliations:** Department of Biomedical Sciences, University of Padua, Padua, Italy

**Keywords:** BIA, BIVA, Keto diet, Phase angle, Very low-carbohydrate ketogenic diet

## Abstract

**Purpose of the Review:**

The use of bioelectrical impedance analysis (BIA) for monitoring body composition during the ketogenic diet has experienced a rapid surge. This scoping review aimed to assess the validity of procedures applying BIA in the ketogenic diet and to suggest best practices for optimizing its utilization.

**Recent Findings:**

We conducted a systematic scoping review of peer-reviewed literature involving BIA for assessing body composition in individuals adhering to a ketogenic diet. Searches of international databases yielded 1609 unique records, 72 of which met the inclusion criteria and were reviewed. Thirty-five studies used foot-to-hand technology, 34 used standing position technology, while 3 did not declare the technology used. Raw bioelectrical parameters were reported in 21 studies. A total of 196 body mass components were estimated, but predictive equations were reported in only four cases.

**Summary:**

Most research on BIA during ketogenic diets did not report the equations used for predicting body composition, making it impossible to assess the validity of BIA outputs. Furthermore, the exceedingly low percentage of studies reporting and analyzing raw data makes it challenging to replicate methodologies in future studies, highlighting that BIA is not being utilized to its full potential. There is a need for more precise technology and device characteristics descriptions, full report of raw bioelectrical data, and predictive equations utilized. Moreover, evaluating raw data through vectorial analysis is strongly recommended. Eventually, we suggest best practices to enhance BIA outcomes during ketogenic diets.

**Supplementary Information:**

The online version contains supplementary material available at 10.1007/s13679-024-00573-0.

## Introduction

### Body Composition Basics

**Fig. 1:**
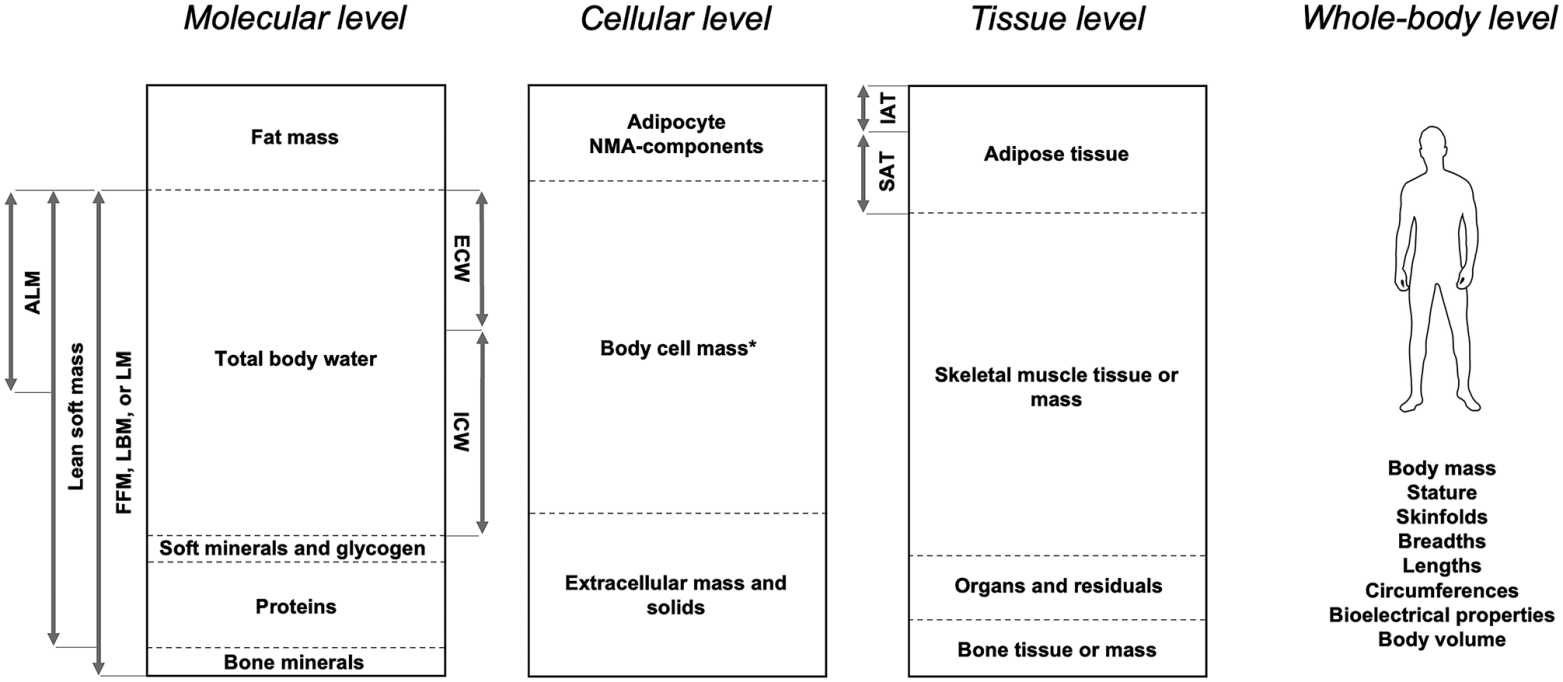
Main body mass components at molecular, cellular, tissue, and whole-body level of body composition. * = Formed by all cells involved in metabolic processes, including the protoplasm in fat cells; FFM= fat-free mass; LBM= lean body mass; LM= lean mass; ALM= Appendicular lean mass; ICW= Intracellular water; ECW= Extracellular water; NMA= nonmetabolically active; IAT= Internal adipose tissue formed by visceral and non-visceral adipose tissue; SAT= Subcutaneous adipose tissue

Body composition describes the components of body mass, which can be organized into five levels in an increasing order of complexity [1]. Starting from the fifth and last level, defined as the whole-body level, dimensional characteristics such as body mass, stature, breadths, lengths, and circumferences allow for a preliminary analysis of body composition. These parameters can be measured and evaluated with inexpensive and easy-to-use tools, such as scales, stadiometers, bone calipers, or measuring tapes. Notable, these dimensions result from the combination of different tissues, whose accurate investigation requires the use of more sophisticated and costly methods, such as imaging techniques [2]. Indeed, the tissue level includes adipose tissue, skeletal muscle mass (SMM), as well as bone tissue, along with other components specific to organs and systems. Each tissue is consisted of different cells immersed in the extracellular fluid, each characterized by a different amount of water inside. Indeed, the percentage of water content varies from around 10% in adipocytes to approximately 80% in myocytes, which is typical for these cell types [3]. Many of these cells engage in metabolic activity, contributing to whole body energy expenditure and oxygen consumption. Collectively, they constitute the body cell mass (BCM), which can be measured using neutron activation techniques [4]. Just as tissues are composed of cells, these cells derive from the combination of various molecules such as lipids, water, minerals, and proteins. At this level, parameters as fat mass (FM), fat-free mass (FFM) or lean body mass (LBM), total body water (TBW), and bone mineral content (BMC) are among the most well-known and monitored in research both for their relationships with health and due to the wide range of tools enabling their analysis [5, 6]. Smaller elements like atoms are part of the first level of body composition and represent the building blocks of any mass. Aside from the atomic level, whose components are challenging to quantify and thus receive limited consideration in research, the following four levels of body composition are illustrated in Fig. 1.

### The Evolution of Bioelectrical Impedance Analysis

**Fig. 2:**
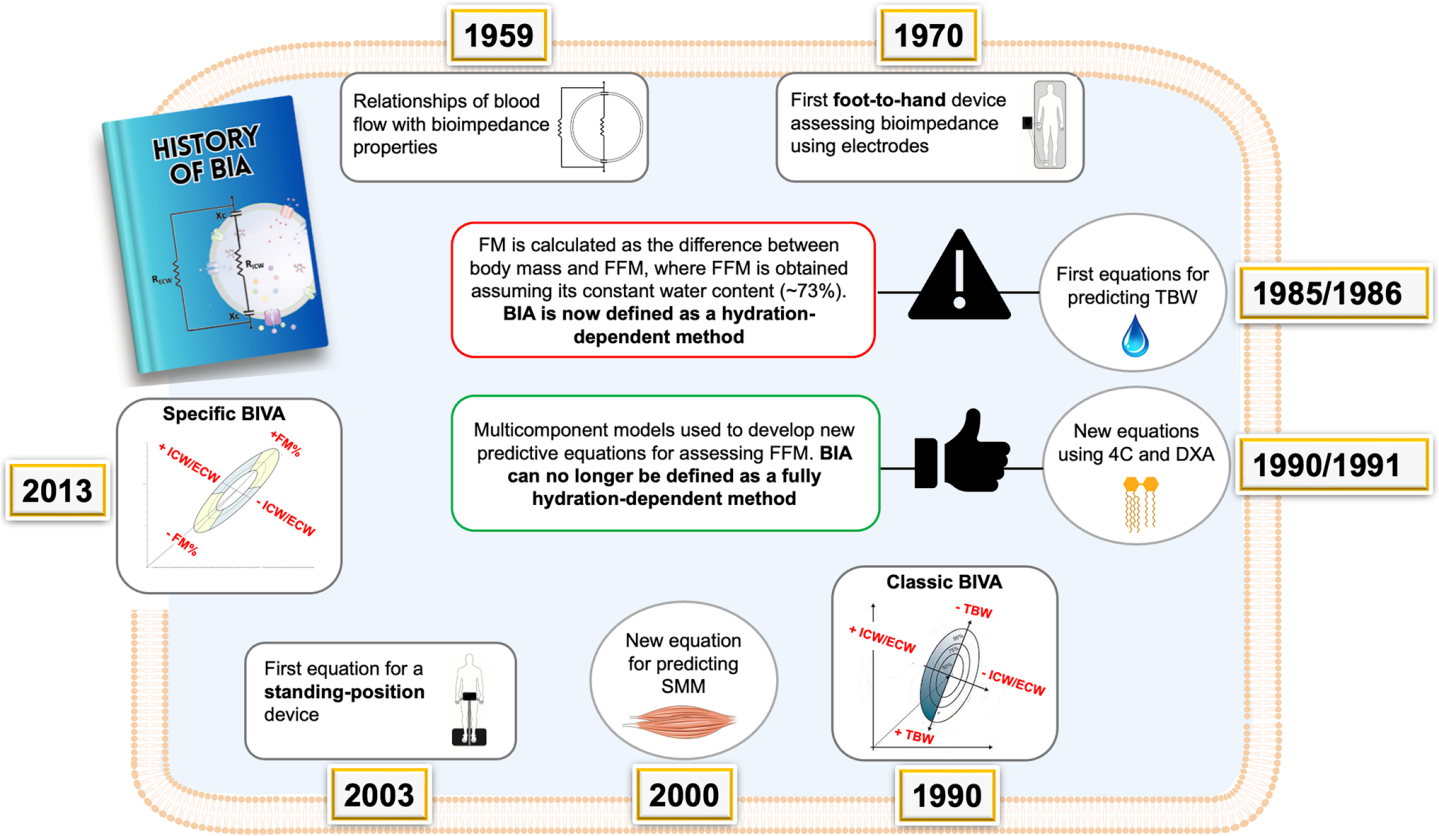
Key events in the history of bioelectrical impedance analysis (BIA). FM: fat mass; FFM: fat-free mass; 4C: four-component model; DXA: dual-energy X-ray absorptiometry; BIVA: bioelectrical impedance vector analysis; ICW: intracellular water; ECW: extracellular water; SMM: skeletal muscle mass

Among the most used tools in scientific research for monitoring body composition is bioelectrical impedance analysis (BIA) [5, 7]. This is due to its ease of use, portability, low cost, and the wide range of parameters it allows for evaluation. BIA is based on the conductive properties of the tissues, where fluids represent the main source of resistance (R) and cellular membranes and other tissue interfaces represent capacitive elements, identified in the reactance (Xc) [4]. The arctangent between R and Xc, calculable as (Xc/R)*(180/π), results in the phase angle (PhA), a biomarker positively associated with the intracellular-to-extracellular water (ICW/ECW) ratio [8, 9]. The history of BIA begins in 1950 when American cardiologist Hans Nyboer discovers the relationship between blood flow and bioelectrical parameters [10]. Subsequently, in 1962, French physician August Luis Thomasset developed the first device to measure bioimpedance in a supine position using a foot-to-hand technology [11]. A few years later, Hoffer et al. (1969) allowed the foot-to-hand technology to evolve into a tetrapolar method by using two pairs of electrodes instead of the two needles previously employed by Thomasset. The clear relationship between bioimpedance and body composition parameters [13] led to the development of the first equations to predict TBW and FFM [14–16]. At this point, FM began to be calculated as the difference between FFM and body mass, even after obtaining FFM under the assumption that it is approximately 73% composed of fluids [17]. These calculations and assumptions, not always valid in all contexts, led to defining BIA as a hydration-dependent method, especially for estimating FM [3, 18]. Subsequently, multicomponent models were used to develop prediction equations for FM, making BIA no longer entirely dependent on hydration status [19, 20]. In 1994, Piccoli et al. [21] suggested that for BIA to achieve high performance in quantifying body composition, a large number of predictive equations specific to age, gender, level of physical activity, and geographical characteristics would be necessary. Alongside this criticism, Piccoli et al. [21] proposed a remedy that involved the analysis of raw data, which he defined as bioelectrical impedance vector analysis (BIVA). In BIVA, R and Xc are standardized based on the subject's stature and considered on the x-axis and y-axis of a Cartesian plane, respectively. The resulting point/vector in representative of the fluid content and its distribution between intra and extracellular compartments [21]. Indeed, the vector length is inversely associated with TBW, while the vector direction graphically represent the PhA [21]. The history of BIA unfolds in the subsequent years, marked by the development of the first equations for predicting skeletal muscle mass (SMM) utilizing magnetic resonance as a reference method [22]. This represented a significant advantage for BIA since FFM broadly encompasses everything not attributable to body fat, thus including a wide variety of components, some of which are metabolically more active (e.g., muscle) than others (bone). In fact, it is known that dosing certain macronutrients would be more appropriate when conducted relative to kilograms of SMM rather than FFM, as in the case of proteins in diets aimed at muscle hypertrophy [23, 24]. Furthermore, there are studies in which SMM increased, even though FFM remained unchanged following specific interventions [25, 26]. This underscores how monitoring FFM alone can mask substantial changes, akin to the situation when observing overall body weight without dissecting it into its relevant components [25]. Advancements in BIA include the production of new device technologies enabling measurements in a standing position, or analyzers integrated into joysticks (hand-to-hand) and home scales (leg-to-leg) [27, 28]. Unfortunately, the use of different technologies results in different outputs, thus preventing the comparison of data obtained from different devices. [29•, 30].While more than 100 equations were available at the end of 2000s [31], BIVA innovation continues with new references for different populations [32••, 33, 34] and a new approach based on standardizing R and Xc on arm, waist, and calf circumferences called *specific* BIVA [35], capable of providing information about FM in addition to the ICW/ECW ratio [9, 36]. Figure 2 summarizes the key events in the history of BIA.

### The Utility of BIA in Ketogenic Diet

The use of BIA is beneficial in all cases where it is necessary to monitor changes in body composition, such as in the context of nutritional interventions. Among the different diets, the ketogenic diet is one of the most controversial that has shown, in the last years, a remarkable surge of scientific studies. Ketogenic diet is a low-carbohydrate, normal protein, high-fat dietary approach designed to induce a metabolic state called ketosis, where the body primarily utilizes fat for energy production [37]. The ketogenic diet (also defined as protein sparing modified fast) [38], was used for the first time as medical therapy by Dr. Wilder in 1921 as dietary approach to epilepsy in alternative to complete fasting [39]. The term “protein sparing modified fast” can be explained by the tentative to reach the metabolic state called ketosis (usually obtained during fasting) without an increased/excessive protein catabolism. In 1926 the first 4:1 ketogenic diet protocol was published [40]; the protocol involved1 g/protein/kg/day, 10 to 15 g/carbohydrates/day, and the remainder of the calories in fat (CHO + PRO: FAT grams, ratio 4:1). This proportion has been substantially maintained along the years [41–44] with some modifications but, in general, the scheme is based on less than 20 /30 g of carbohydrates or less than 5% of total daily energy intake from carbohydrate [45]. Recently, a clear classification of different types of ketogenic diets has been published [46]. In the recent year the interest about Ketogenic diet exceeded the limits of the original use in epilepsy and moved to include other clinical conditions such as inflammation [47, 48], metabolic syndrome, type 2 diabetes, polycystic ovary syndrome, cancer and others not communicable diseases [37]. The ketogenic diet is emerging as a cornerstone in public health strategies and interventions, given its potential to swiftly mitigate all modifiable risk factors, particularly addressing the complex issue of obesity and its associated metabolic sequelae [49••].

The suggested positive effects exerted by the ketogenic diet on health, relay on the “physiological ketosis” described by Hans Krebs in 1966 [50] who remarked the differences from the pathological ketosis (e.g., in decompensated diabetes [51]). Whilst the pathological ketosis is characterized by low systemic pH, no insulin, hyperglycaemia and very high level of ketone bodies (> 7/8 mmol/L), the physiological one has normal pH, low but within physiological ranges of both insulin and blood glucose. The levels of ketone bodies, produced from fats by the liver during carbohydrate restriction, varies typically between 0.3 up to 4 mmol/L [37, 44]. As said, ketone bodies are produced mainly in the liver (ketogenesis) from Acetyl-CoA. The condensation of two acetyl CoA leads to the production of an acetoactylCoa in a reaction catalyzed by the acetoacetyl-CoA thiolase, then the acetoacetyl Coa becomes 3HMGCoA through the addition of a third acetyl-CoA (catalyzed by the mitochondrial HMG-CoA synthetase). The HMG-CoA lyase catalyzes the passage from HMG-CoA to acetoacetate plus acetyl-CoA. Acetoacetate is the first ketone body which can be reduced by the NADH-dependent β-hydroxybutyrate dehydrogenase into the other ketone body: the β-Hydroxybutyrate and, by a spontaneous decarboxylation, to acetone, the third, volatile ketone. ketone bodies are not merely a byproduct of a CHO restricted condition but act as metabolic agents on different pathways [52]. One of the main fields of ketogenic diets’ use is weight control or weight loss and relatively changes of body composition variables. Considering fluids, the ketogenic diet typically leads to a significant reduction in carbohydrate intake, which in turn causes the body to release stored glycogen. Glycogen is stored with water, so as glycogen is depleted, the body also loses water weight. This initial loss of fluids is often noticeable, and monitoring fluid changes helps individuals understand short-term shifts in weight and hydration status. Moreover, during low carbohydrate availability periods, preserving skeletal muscle mass is a key concern for individuals. In fact, monitoring changes in SMM helps individuals ensure that their weight loss is predominantly from fat, rather than muscle. Given the primary body composition parameters of interest in ketogenic diet research, the utilization of BIA is progressively on the rise within this particular context [53].

### Aim of the Study

Recently, it has been pointed out that BIA is not always applied with the correct procedures or fully exploited to its potential [28, 32••, 54]. This is due to the rapid evolution of BIA and the numerous innovations stemming from various studies that would need to be summarized. The list of procedures that delineate a proper BIA begins with an extensive description of device technology and the raw measured parameters. These procedures require the selection of specific predictive equations tailored to the subject under examination, and reporting them is crucial not only for ensuring the validity of the outputs but also for enabling future comparisons, given that each equation can yield different results [28, 31]. For these reasons, evaluating raw parameters through BIVA, in conjunction with body composition estimations, can facilitate a more comprehensive and qualitative analysis [32••, 55]. Therefore, the objective of this review was to systematically select all studies in which BIA has been used to analyze body composition in ketogenic diet interventions and consider whether the methods applied can be considered satisfactory. The hypothesis underlying this scoping review was that the BIA procedures might have shortcomings or that it had not always been utilized to its full capacity. Given the potential of BIA in evaluating parameters of particular interest to the ketogenic diet, the secondary goal was to define best practices to optimize its use, ensuring the validity of BIA-based assessments.

## Methods

### Search Strategy

The present study was carried out following the Preferred Reporting Items for Systematic reviews and Meta-Analyses Extension for Scoping Reviews (PRISMA-ScR) guidelines [56]. The literature search was carried out by consulting the main biomedical databases such as Pubmed (Medline), and Scopus on Dec 31st, 2023, identifying potential eligible studies without any restriction related to year of publication. We composed different query strings depending on the variability of the databases functioning, using the following terms: ((ketogenic diet) OR (keto diet)) AND ((body composition) OR (fat mass) OR (body fluids) OR (total body water) OR (extracellular water) OR (intracellular water) OR (FM) OR (TBW) OR (ECW) OR (ICW) OR (bioelectrical impedance analysis) OR (bioimpedance) OR (BIA)). To ensure the inclusion of all potentially relevant sources, supplementary records were searched through cross-referencing.

### Eligibility Criteria

Articles were included in the review if they met the following inclusion criteria: i) using BIA for assessing body composition in keto diet (< 50 g/d, or < 10% of daily energy intake) in humans; ii) accessible in English full text. Exclusion criteria were research protocols, thesis/dissertations, abstracts, letters to the editor, case reports, reviews, book chapters, guidelines, position papers, and unpublished works. Articles that did not meet the eligibility criteria were excluded via screening title, abstract, and full-text review.

### Data Extraction

Based on the initial titles retrieved, duplicates were removed. After concluding the search, all records were compiled into the Endnote for Windows version X9, 2018 (Clarivate, Philadelphia, USA) software to delete all duplicates showing the same: a) title, authors and year of publication and b) title, authors, and journal title. The records remaining after the deletion of duplicates were exported to an Excel file for Windows version 16.75.2 (Microsoft, Washinton, EUA) organized based on essential information for screening, such as authors’ names, publication year, journal title, digital object identification (DOI), article title and abstract. Abstracts identified from the literature searches were screened for potential inclusion by two authors (F.C. and A.P.). The major characteristics extracted from the included studies were year of publication, study design and duration, sample size, BIA technology and device, bioelectrical measures and body composition estimations, and characteristics of the predictive equations used for estimating body composition.

## Results

**Fig. 3:**
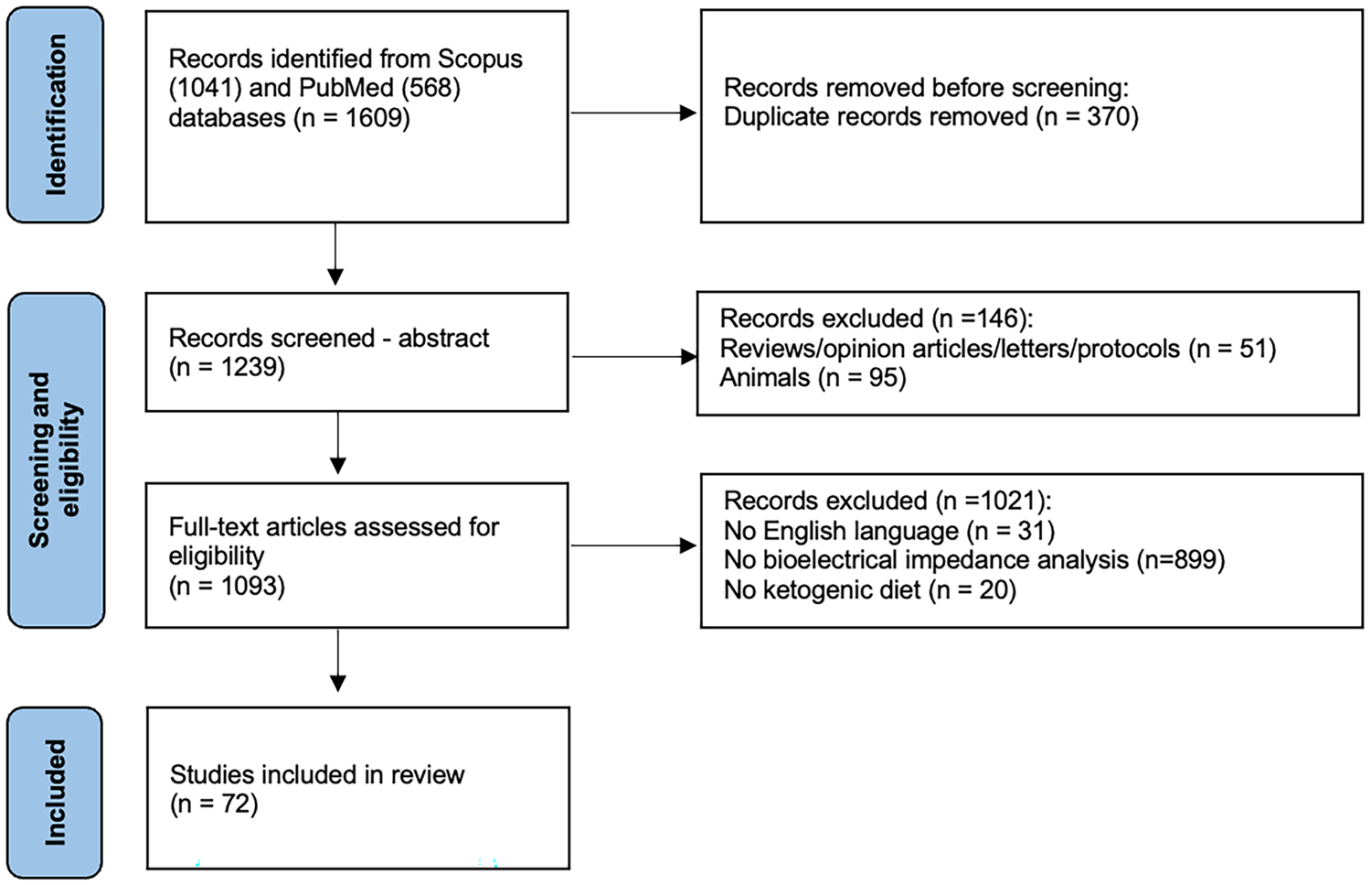
PRISMA flow chart for the search strategy

The seventy-two selected studies involved 3,825 participants (age ranging 2 – 78 y, body mass index ranging 15.3 – 50.9 kg/m^2^) of both genders. The procedure of the systematic search and the characteristics of the included studies are reported in Fig. 3 and Supplementary Table 1, respectively.

### Technologies and Bioelectrical Raw Data

**Fig. 4:**
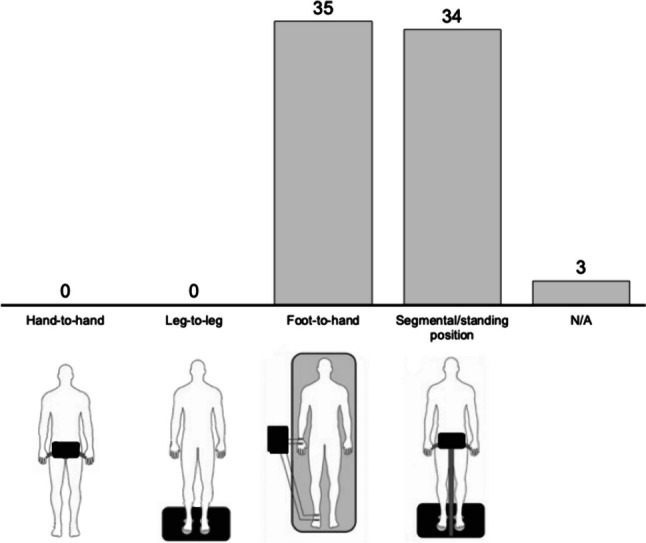
Number of bioimpedance technologies used in the selected studies (N = 72)

The layout of measurements for each of the four bioimpedance technologies is illustrated in Fig. 4, along with the corresponding number of studies that utilized them. Thirty-five studies employed a foot-to-hand technology [17, 57–63, 64•, 65–89], 34 utilized a standing position/segmental technology [90–124], and 3 did not report any information about the technology used [117, 125, 126]. No study employed the hand-to-hand or leg-to-leg technology. Five studies reported raw bioelectrical parameters of R, Xc, and PhA [64•, 73, 81, 82, 89], three reported only R and Xc [17, 65, 83], and 12 reported only PhA [61, 69, 71, 72, 80, 85, 86, 98, 99, 103, 105, 127].

### Prediction of Body Mass Components

**Fig. 5:**
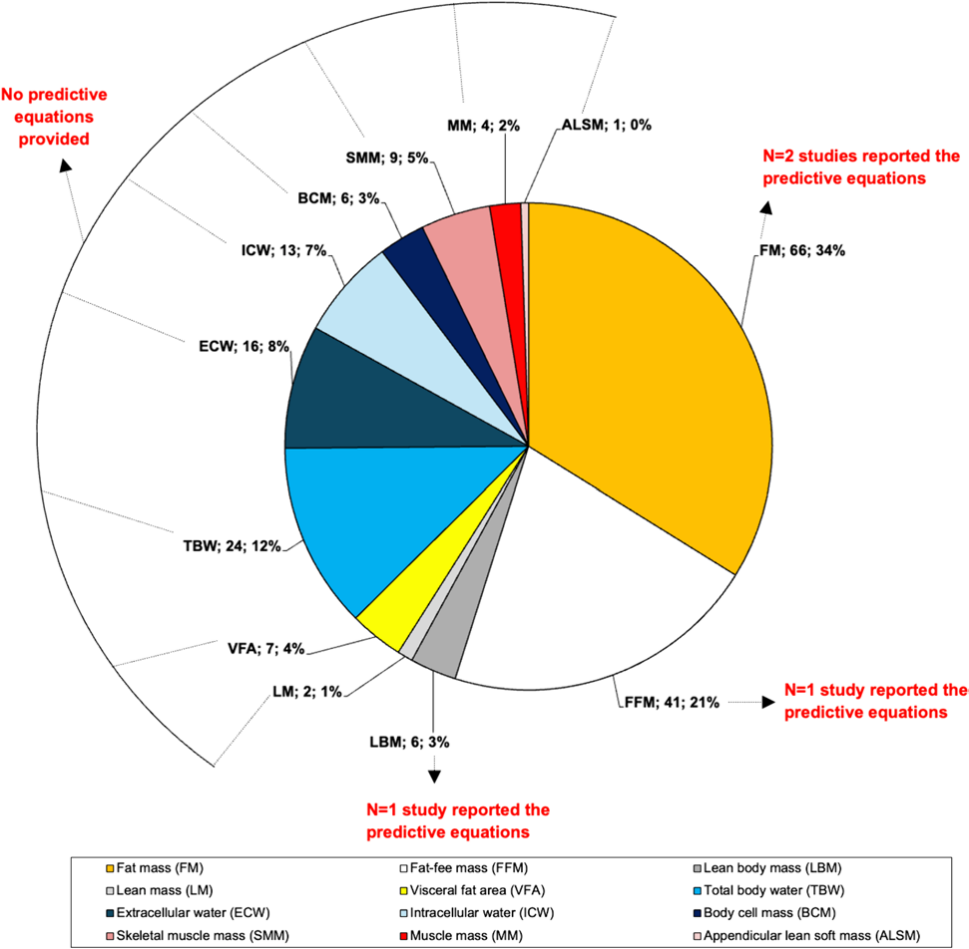
Pie chart illustrating the number of predicted body composition variables and their respective percentages in relation to the total predictions (N = 192) across the selected studies (N = 72)

Figure 5 shows the predicted variables in the 72 included studies, indicating for each variable the number of studies in which it was considered and the percentage relative to the total predictions (N = 195).

Out of the 72 studies, 66 predicted FM [57–61, 63, 65–69, 71–85, 87, 88, 90–127], 41 predicted FFM [57–59, 63, 65–67, 69, 73–78, 80–82, 84, 85, 88, 90, 93, 98–101, 103, 105, 108–110, 114, 115, 117, 118, 120–124, 127], 24 predicted TBW [57, 58, 62, 69–72, 84, 89, 98–101, 103, 105, 108–110, 114–116, 118, 120, 121], 16 predicted ECW [58, 62, 69–72, 78, 89, 98, 99, 101, 103, 109, 118, 120, 122], 13 predicted ICW [58, 62, 69, 71, 78, 98, 99, 101, 103, 109, 118, 120, 121], 9 predicted SMM [58, 88, 96, 100, 107, 109, 110, 115, 121], 7 predicted visceral fat area [104, 107, 112, 115, 117, 121, 124], 6 predicted BCM [61, 67, 69, 85, 86, 89] and lean body mass [17, 79, 95, 106, 111, 126], 3 predicted muscle mass [115, 116, 122], 2 predicted lean mass [71, 94], and 1 predicted appendicular muscle mass [121],. Predictive equations have been reported in only three studies for the estimation of FM [76, 87], FFM [76], and LBM [17]. Ferraris et al. (2019) estimated FM using the following equation provided by Houtkooper et al. (1992): FM = body mass – FFM, where FFM = 0.58 * (R) + 0.24 * (weight) + 2.69. Pandurevic et al. (2023) estimated FM and FFM used the equation developed by Sun et al. (2003): FM = Weight – FFM, where FFM =  − 10.68 + 0.65 × (stature^2^/R) + 0.26 × body mass + 0.02 × R. Vazquez and Janosky (1991) estimated LBM using eight different equations: 1) LBM = 0.6483 * (stature)^2^ + 0.1699* (body mass) + 5.091, by Segal et al. (1985); 2) LBM = 0.21411 * (stature) + 0.36273 * (stature^2^/R) + 0.1329 * (body mass) – 5.61911 * gender (where 1 is for female and 0 for male) – 8.98751, by Segal et al. (1985); 3) LBM = 0.821 * (stature^2^/R) + 4.917, by Lukaski et al. (1986); 4) LBM = 0.00085 * stature^2^—0.02375 * R + 0.3736 * (body mass)—0.1531 * (age) – 4.2921 * gender (where 1 is for female and 0 for male) + 17.7868, by Van Loan et al. (1987); 5) LBM = 0.00091186 * (stature)^2^—0.01466 * (R) + 0.29990 * (body mass)—0.07012 * (age) + 9.37938, by Segal et al. (1988); 6) LBM = 0.000985 * (stature)^2^—0.0387* (R) + 0.158 * (body mass) – 0.124 * (age) + 29.612, by Gray et al. (1989); 7) LBM = 0.698 * (stature^2^/R) + 3.5 * gender (where 0 is for female and 1 for male) + 9.4, by Deurenberg et al. (1989); 8) LBM = TBW/0.73, where TBW = 0.382 * (stature^2^/R) + 0.105 * (body mass) + 8.315, by Kushner et al. (1990).

## Discussion

BIA enables the assessment of key body composition parameters relevant to the ketogenic diet [53, 136]. The main purpose of this scoping review was to select all studies that utilized BIA in ketogenic diet programs, assessing the validity of the procedures employed in analyzing body composition. A second aim was to suggest best practices for fully leveraging the potential of this technique. The results indicated that methods applied to BIA’s use are, generally, not well described, undermining the validity of the extracted data. This arises from the high number of studies that have not reported the prediction formulas used to estimate body composition, and the few compliant with this first point did not always used specific formulas suitable for the participants under examination. Moreover, few studies directly evaluated raw parameters, and none of them utilized *classic* or *specific* BIVA to monitor body fluids, their distribution, as well as FM.

### Technologies and Bioelectrical Raw Data

BIA can currently be performed using four different types of devices, categorized into four distinct technologies. The studies included in this review have employed foot-to-hand and standing position, also known as segmental technology, while no study has utilized hand-to-hand and leg-to-leg technologies. However, three studies did not declare the technology used, nor the name of the device [117, 125, 126]. Reporting these types of information is relevant for comparison purposes, given the lack of agreement between the absolute data measured by different technologies [29•, 30]. Moreover, only 21 out of 72 studies reported raw data measured with BIA. Double-indirect methods for body composition analysis use raw measurements, such as R, Xc, and PhA, within regression models to estimate body mass components [3]. Hence, it is advisable to consistently include raw measurements, as they constitute the independent variables necessary for predictive models. Moreover, these raw data can be evaluated independently without necessarily being incorporated into predictive equations. For example, PhA has proven to be a reliable biomarker of fluid distribution between compartments, as well as being correlated with cellular integrity and density [64•]. By monitoring the phase angle, it's possible to qualitatively assess any compromise or improvement in cellular integrity, which plays a crucial role in determining muscle quality [64•]. Furthermore, through BIVA, it is possible to monitor PhA, representing the vector's distance from the x-axis, along with vector length which is a strong predictor of TBW [137] in *classic* BIVA [32••] or FM in *specific* BIVA [138]. This could be particularly useful in ketogenic diets to monitor glycogen depletion leading to intracellular water loss [49••]. Additionally, one of the goals during a ketogenic diet is to avoid excessive loss of SMM, a component primarily formed by fluids, especially intracellular ones [53]. In this regard, leftward vector displacements within the R-Xc graph identify increases in the ICW/ECW and then in SMM for both BIVA approaches [5]. Evaluating raw parameters without relying on predictive equations could help avoid dependence on estimation errors inherent in regression models. Such errors are even more pronounced when formulas unsuitable for the subject at hand are used, and unfortunately, there are still no formulas available for all populations to date [32••].

### Prediction of Body Mass Components

A total of 196 estimates for various body mass components resulted from the 72 studies included in this review. However, only 4 studies reported the predictive formulas used, allowing for their discussion. Vasquez and and Janosky (1991) used eight different equations for estimating LBM. Considering that participants were adult females with obesity (Supp. Table 1) the use of the four formulas [130–132] appeared to be inappropriate since they were developed on normal weight females. One of the 8 formulas, instead, was developed on overweight subjects but mixed adults and elderly individuals [134]. It is worth noting that one equation estimated FFM from TBW, assuming that it can be composed of 73% fluids, a condition that is not always valid, especially in obese subjects who may exhibit overhydration [139]. In contrast, the other two formulas involved in the same study [17] may be valid since they were developed including obese subjects [15, 133]. Ferraris et al. [87] included participants aged 2 to 17 years and used the equation provided by Houtkooper et al. [128],which is developed on subjects similar age. Finally, Pandurevic et al. [76] considered adult female subjects and used the made by Sun et al. [129], which suffers from having been developed by mixing different populations (under 18, adults, and elderly people). In the remaining 68 studies, where the utilized predictive equations were not disclosed, evaluating the validity of the regression models concerning the characteristics of the involved participants becomes unfeasible. This is because the predictive equations, in addition to being specific to technologies, are also population-specific, and the use of different formulas can yield different results for the same parameter [17]. To date, a wide range of predictive equations is available in the literature, especially for the parameters that were most considered in the 72 studies, such as FM, FFM, fluids, SMM, BCM, and ALSM [140–149]. Conversely, to our knowledge, no equations are available for VFA and the algorithms used by some studies may be proprietary to the manufacturers. Moreover, the term muscle mass was incorrectly used interchangeably with FFM. Therefore, reporting the predictive equations used and adhering to correct terminology in accordance with the body composition basics (Fig. 1) not only allows future studies to replicate the procedures but also provides physicians and nutritionists with reference data in the clinical context.

### Best Practices for Using BIA in Ketogenic Diet

In light of the above, the following best practices are discussed for improving the use of BIA in ketogenic diet studies:

- Technology and device characteristics: these should always be reported because each device provides measurements that are not comparable with other technologies [29•].
- Raw BIA data: reporting raw data allows for their use in new predictive equations or as reference data. Additionally, the assessment of PhA provides an indication of fluid distribution among compartments. Increases in PhA identify increases in the ICW/ECW ratio and vice versa [8, 64•].
- Predictive equations: the equations should be reported among the methods and their selection should be justified. They are specific to technology and should be developed on subjects with similar characteristics to those being tested. Currently, there are various predictive equations for both the general population (under 18, adults, and elderly people) and athletes, developed for a wide range of body composition parameters and versus multicompartmental models using gold standard procedures [6, 150••].
- Raw data analysis through *classic* or *specific* BIVA: when valid prediction equations are not available in the literature, the use of BIVA may avoid systematic estimation errors. Furthermore, reference tolerance ellipses to incorporate into R-Xc graphs are available for the general population and athletes [9, 32••].

**Fig. 6:**
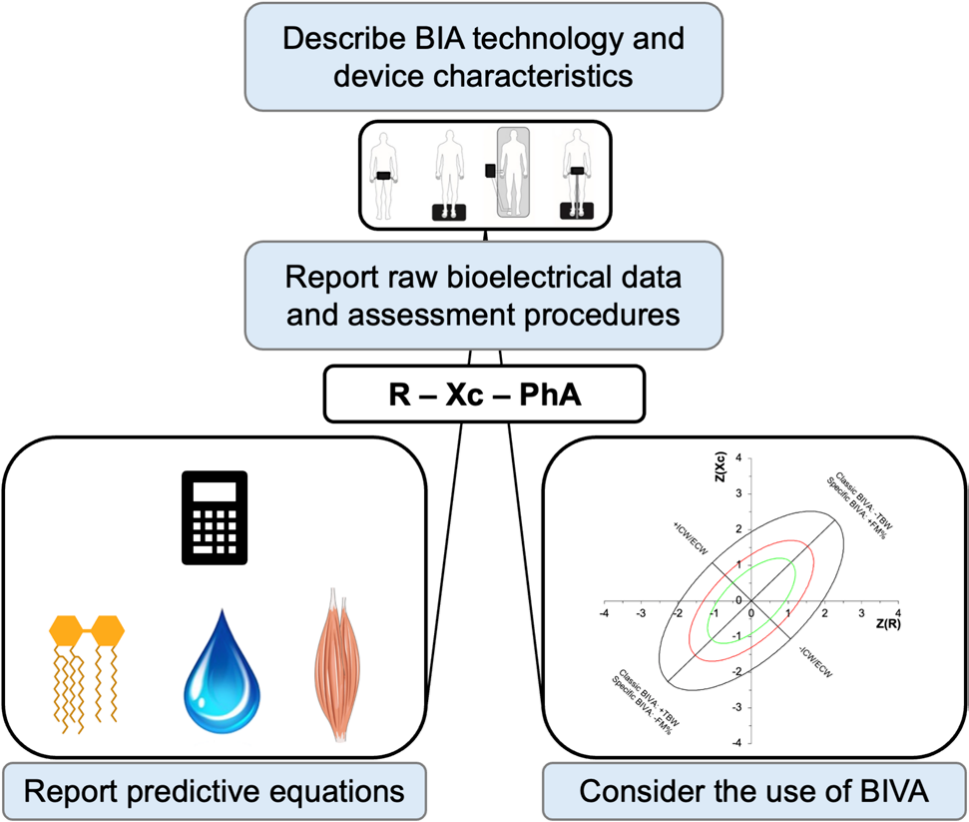
Best practices for studies using BIA

The suggested best practices are schematized in Fig. 6.

### Review Limitations and Future Perspectives

Some limitations about this review should be mentioned. Data on changes in body composition were not extracted. However, as hypothesized, the almost complete absence of information on the predictive equations used in the studies does not allow for certainty regarding the validity of the body composition outputs. Therefore, future systematic reviews will be necessary to analyze changes in body composition on a bioelectrical basis when enough studies have adopted best practices in its use during ketogenic diet. Another limitation is the failure to provide a list of predictive equations for each technology and each body composition variable. However, to date, such formulas are widespread in the literature, and no study has collected and cataloged them for different independent factors such as age, sex, health status, and level of physical activity. In this regard, a systematic review should be conducted to categorize all BIA-based prediction equations, facilitating their retrieval in the literature.

## Conclusions

Until now, BIA has been employed in studies examining the efficacy of the ketogenic diet using methodologies with some limitations. The primary concern is the almost complete absence of descriptions (predictive equations) of the procedures used to estimate body composition. This lack of information compromises the validity of the obtained data and hinders future comparisons. Moreover, the majority of studies do not provide raw parameters, forfeiting the opportunity to analyze them qualitatively, thus avoiding potential intrinsic estimation errors in quantitative analysis of body mass components. We tried to suggest some best practices to enhance the use of BIA during ketogenic diet interventions, encompassing the description of BIA technologies and raw data, the selection of predictive equations, and the adoption of alternative analyses such as BIVA, which allows for qualitative monitoring of fluids and body fat.

### Supplementary Information

Below is the link to the electronic supplementary material.

Supplementary file1 (DOCX 43 KB)

## Data Availability

Not applicable.

## Abbreviations

BCM: Body cell mass
BMC: Bone mineral content
BIA: Bioelectrical impedance analysis
BIVA: Bioelectrical impedance vector analysis
CHO: Carbohydrate
ECW: Extracellular water
FM: Fat mass
FFM: Fat-free mass
ICE: Intracellular water
LBM: Lean body mass
LM: Lean mass
LSM: Lean soft mass
PhA: Phase angle
PRO: Protein
R: Resistance
SMM: Skeletal muscle mass
TBW: Total body water
VFA: Visceral fat area
Xc: Reactance
