# Supplementary material for: Problems and Opportunities in the use of Bioelectrical Impedance Analysis for Assessing Body Composition During Ketogenic Diets: A Scoping Review
Source: Curr Obes Rep. 2024 May 27;13(3):496–509. doi: 10.1007/s13679-024-00573-0 (PMC11306364; doi:10.1007/s13679-024-00573-0)
Supplement: Supplementary file 1 — Supplementary file1 (DOCX 43 KB) [file 13679_2024_573_MOESM1_ESM.docx]

**The use of bioelectrical impedance analysis for assessing body composition during ketogenic diets: problems and opportunities - a scoping review**.

*Current Obesity Reports*

Antonio Paoli 1,2*and Francesco Campa1

1 Department of Biomedical Sciences, University of Padua, Padua, Italy

2 Research Center for High Performance Sport, UCAM, Catholic University of Murcia, 30107 Murcia, Spain.

* Correspondence: Antonio Paoli, MD

E-mail address: antonio.paoli@unipd.it

University of Padova

Via 8 Febbraio 1848, 2

35122 Padova, Italy

phone: +39 0498273034

| **Table 1**. Characteristics of the included studies (N= 72). | | | | | | |
| --- | --- | --- | --- | --- | --- | --- |
| Authors | Year | Participants | Design | Analyzer | BIA-outcomes | Procedures |
| Vasquez et al. | 1991 | N= 8 females, age 44 (42) y, BMI 47 (2) kg/m^2^ | Longitudinal, 4 weeks | Technology: foot-to-hand  Frequency: 50 kHz  Device: BIA-103, RJL | Raw measures:  Z, R, and Xc  Estimates:  LBM | LBM estimated using eight predictive equations:   - LBM = 0.6483 * (height)^2^ + 0.1699* (weight) + 5.091, by Segal et al. (1985) - LBM = 0.21411 * (height) + 0.36273 * (height^2^/R) + 0.1329 * (weight) – 5.61911 * (1 for female or 0 for male) – 8.98751, by Segal et al. (1988) - LBM = 0.821 * (height^2^/R) + 4.917, by Lukaski et al. (1986) - LBM = 0.00085 * (height)^2^ - 0.02375 * (R) + 0.3736 * (weight) - 0.1531 * (age) – 4.2921 * (1 for female or 0 for male) + 17.7868, by Van Loan et al. (1987) - LBM = 0.00091186 * (height)^2^ - 0.01466 * (R) + 0.29990 * (weight) - 0.07012 * (age) + 9.37938, by Segal et al. (1988) - LBM = 0.000985* (height)^2^ - 0.0387* (R) + 0.158 * (weight) – 0.124 * (age) + 29.612, by Gray et al. (1989) - LBM = 0.698 * (height^2^/R) + 3.5 * (0 for female or 1 for male) + 9.4, by Deurenberg et al. (1989) - LBM = TBW/0.73; TBW = 0.382 * (height^2^/R) + 0.105 * (weight) + 8.315, by Kushner et al. (1990) |
| Yancy et al. | 2004 | N= 34 males (22%) and females (78), age 44.1 (8.7) y, BMI 33.9 (5.3) kg/m^2^ | Longitudinal, 24 weeks | Technology: segmental  Frequency: multifrequency  Device: TBF-300A, Tanita Co. | Raw measures:  N/A  Estimates:  FM and FFM | Predictive equations for estimating body composition were not reported |
| Johnston et al. | 2006 | N= 2 males and 7 females, age 38.4 (3.9) y, BMI 35 (1.6) kg/m^2^ | Longitudinal, 6 weeks | Technology: segmental  Frequency: multifrequency  Device: TBF-300A, Tanita Co. | Raw measures:  N/A  Estimates:  FM | Predictive equations for estimating body composition were not reported |
| Nazarewicz et al. | 2007 | N= 20 females, age from 21-23y, BMI 21.5 (2.1) kg/m^2^ | Longitudinal, 14 days | Technology: N/A  Frequency: N/A  Device: N/A | Raw measures:  N/A  Estimates:  FM | Predictive equations for estimating body composition were not reported |
| White et al. | 2007 | N= 2 males and 8 females, age 38.4 (3.9) y, BMI 34.6 (1.6) kg/m^2^ | Longitudinal, 2 weeks | Technology: segmental  Frequency: multifrequency  Device: TBF-300A, Tanita Co. | Raw measures:  N/A  Estimates:  FM | Predictive equations for estimating body composition were not reported |
| Paoli et al. | 2010 | N= 10 females and 7 males, age 41.4 (8) y, BMI 29.8 (3.5) kg/m^2^ | Longitudinal, 6 weeks | Technology: foot-to-hand  Frequency: 50 kHz  Device: BIA-101, Akern | Raw measures:  N/A  Estimates:  FM, FFM, and TBW | Predictive equations for estimating body composition were not reported |
| Paoli et al. | 2011 | N= 87 females and 19 males, age 48.5 (10.3) y, BMI >25 kg/m^2^ | Longitudinal, 6 weeks | Technology: foot-to-hand  Frequency: 50 kHz  Device: BIA-101, Akern | Raw measures:  N/A  Estimates:  FM | Predictive equations for estimating body composition were not reported |
| Partsalaki et al. | 2012 | N= 15 females and 14 males, age 13.6 (2.5) y, BMI 30.8 (8.1) kg/m^2^ | Longitudinal, 6 months | Technology: segmental  Frequency: multifrequency Device: TBF-300A, Tanita Co. | Raw measures:  N/A  Estimates:  FM | Predictive equations for estimating body composition were not reported |
| Rhyu et al. | 2012 | N= 10 athletic subjects, age 16.4 (0.9) y, BMI 21.4 (2.1) kg/m^2^ | Longitudinal,  3 weeks | Technology: N/A  Frequency: N/A  Device: N/A | Raw measures:  N/A  Estimates:  FM and LBM | Predictive equations for estimating body composition were not reported |
| Zajac et al. | 2014 | N= 8 male cyclists, age 28.3 (3.9) y, BMI 24.8 (3.1) kg/m^2^ | Longitudinal,  4 weeks | Technology: segmental  Frequency: multifrequency  Device: Inbody 720, Biospace Co. | Raw measures:  N/A  Estimates:  FM | Predictive equations for estimating body composition were not reported |
| Paoli et al. | 2015 | N= 38 subjects, age 56.3 (5.1), BMI 29.3 (2.4) | Longitudinal,  4 weeks | Technology: foot-to-hand  Frequency: 50 kHz  Device: BIA-101, Akern | Raw measures:  N/A  Estimates:  FM and LBM | Predictive equations for estimating body composition were not reported |
| Rubini et al. | 2015 | N= 16 subjects, age 51.4 (12.4), BMI 29.3 (2.4) kg/m^2^ | Longitudinal,  20 days | Technology: foot-to-hand  Frequency: 50 kHz  Device: BIA-101, Akern | Raw measures:  N/A  Estimates:  FM, FFM, and TBW | Predictive equations for estimating body composition were not reported |
| Klement and Sweeney | 2016 | N= 6 oncologic subjects (2 females), age 40-74 y, BMI 20.6 – 28.5 kg/m^2^ | Longitudinal,  32-73 days | Technology: segmental  Frequency: multifrequency  Device: mBCA 515/514, SECA | Raw measures:  PhA at 50 kHz  Estimates:  FM, FFM, TBW, ECW, and ICW | Predictive equations for estimating body composition were not reported |
| Gomez-Arbelaez et al. | 2017 | N= 20 subjects age, 47.2 (6 10.2) y, BMI,  35.5 (4.4) kg/m^2^ | Longitudinal,  4 months | Technology: segmental  Frequency: multifrequency  Device: Inbody 720, Biospace Co. | Raw measures:  N/A  Estimates:  FM, FFM, VFA, TBW, ECW, ICW, SMM, ALSM | Predictive equations for estimating body composition were not reported |
| Urbain et al. | 2017 | N= 31 females and 11 males, mean age 37 (12), mean BMI 23.9 (3.1) kg/m^2^ | Longitudinal,  4 weeks | Technology: foot-to-hand  Frequency: multifrequency  Device: Nutriguard-M, Data Input GmbH | Raw measures:  PhA at 50 kHz  Estimates:  FM, FFM, and BCM | Predictive equations for estimating body composition were not reported |
| Gomez-Arbelaez et al. | 2018 | N= 12 females and 8 males age 18-58 y, BMI,  35.5 (4.4) kg/m^2^ | Longitudinal,  4 months | Technology: segmental  Frequency: multifrequency  Device: Inbody 720, Biospace Co. | Raw measures:  N/A  Estimates:  FM, FFM, and MM | Predictive equations for estimating body composition were not reported |
| Pilone et al. | 2018 | N= 75 females and 44 males, age 43.6 (9.8) y, BMI 41.5 (7.6) kg/m^2^ | Longitudinal,  10 days | Technology: segmental  Frequency: multifrequency  Device: MC-180-MA, Tanita Co. | Raw measures:  N/A  Estimates:  FM, FFM, and VFA | Predictive equations for estimating body composition were not reported |
| Cannataro et al. | 2019 | N= 18 females, age 46.8 (12.4), BMI > 25 kg/m^2^, and 18 males, age 46.4 (10.5), BMI > 25 kg/m^2^ | Longitudinal,  4 weeks | Technology: foot-to-hand  Frequency: multifrequency  Device: DF50, ImpediMED | Raw measures:  PhA at 50 kHz  Estimates:  BCM | Predictive equations for estimating body composition were not reported |
| Ferraris et al. | 2019 | N= 34 subjects, age 2-17 y, BMI 15.3-19.4 kg/m^2^ | Longitudinal,  12 months | Technology: foot-to-hand  Frequency: multifrequency  Device: Handy 3000, DS-Medica | Raw measures:  N/A  Estimates:  FM | FM= Weight – FFM; FFM= 0.58 * (R) + 0.24 * (weight) + 2.69, by Houtkooper et al. 1992 |
| Ministrini et al. | 2019 | N= 52 subjects, age 18-65 y, BMI 44.7 (8.3) kg/m^2^ | Longitudinal, 25 days | Technology: segmental  Frequency: 50 kHz  Device: TBF-410GS, Tanita Co. | Raw measures:  N/A  Estimates:  FM | Predictive equations for estimating body composition were not reported |
| Mohorko et al. | 2019 | N= 13 males, age 38 (7) y, BMI 38 (6) kg/m^2^, and 25 females age 36 (6) y, BMI 35 (5) kg/m^2^ | Longitudinal,  12 weeks | Technology: segmental  Frequency: multifrequency  Device: TBF-410GS, Tanita Co. | Raw measures:  N/A  Estimates:  FM and FFM | Predictive equations for estimating body composition were not reported |
| Perticone et al. | 2019 | N= 14 males and 14 females, mean age 46.8 (11) y, mean BMI 40.5 (10.1) kg/m^2^ | Longitudinal,  12 months | Technology: foot-to-hand  Frequency: 50 kHz  Device: BIA-101, Akern | Raw measures:  N/A  Estimates:  FM, FFM, and SMM | Predictive equations for estimating body composition were not reported |
| Prins et al. | 2019 | N= 7 males, age 35.6 (8) y, BMI 21.5 (1.1) kg/m^2^ | Longitudinal,  6 weeks | Technology: segmental  Frequency: multifrequency  Device: MC-980Uplus, Tanita Co. | Raw measures:  N/A  Estimates:  FM, and LM | Predictive equations for estimating body composition were not reported |
| Romano et al. | 2019 | N= 10 males and 10 females, mean age 56.1 (9.3), mean BMI 37.1 (6.8) kg/m^2^ | Longitudinal,  8 weeks | Technology: foot-to-hand  Frequency: 50 kHz  Device: BIA-101, Akern | Raw measures:  R, Xc, and PhA  Estimates:  TBW, ECW, and BCM | Predictive equations for estimating body composition were not reported |
| Bruci et al. | 2020 | N= 69 females and 23 males, mean age 51.3 (12.2), mean BMI 33.9 (5.8) kg/m^2^ | Longitudinal, 3 months | Technology: foot-to-hand  Frequency: 50 kHz  Device: BIA-101, Akern | Raw measures:  N/A  Estimates:  FM, FFM, TBW, ECW, ICW, and SMM | Predictive equations for estimating body composition were not reported |
| Buechert et al. | 2020 | N= 12 subjects, age 48.4 (11.3), BMI 25.8 (2.8) kg/m^2^ | Longitudinal, 6 weeks | Technology: foot-to-hand  Frequency: multifrequency  Device: 2000-M, Data Input | Raw measures:  N/A  Estimates:  FM, and FFM | Predictive equations for estimating body composition were not reported |
| D’Abbondanza et al. | 2020 | N= 42 females, age 17-67 y, BMI 46 (8) kg/m^2^, and 28 males, age 20-62 y, BMI 48 (7) kg/m^2^ | Longitudinal, 25 days | Technology: segmental  Frequency: multifrequency  Device: TBF-410GS, Tanita Co. | Raw measures:  N/A  Estimates:  FM, and FFM | Predictive equations for estimating body composition were not reported |
| Hadizadeh et al. | 2020 | N= 10 subjects age 35.2 (10.1) y, BMI 23.6 (4.6) kg/m^2^ | Longitudinal, 8 weeks | Technology: segmental  Frequency: multifrequency  Device: Inbody 570, Biospace Co. | Raw measures:  N/A  Estimates:  FM, and LBM | Predictive equations for estimating body composition were not reported |
| Hagihara et al. | 2020 | N= 22 females and 15 males, mean age 54 (12.6) y, mean BMI 20.9 (3.7) kg/m^2^ | Longitudinal, 3 months | Technology: segmental  Frequency: multifrequency  Device: Inbody 720, Biospace Co. | Raw measures:  N/A  Estimates:  FM, and SMM | Predictive equations for estimating body composition were not reported |
| Khodabakhshi et al. | 2020 | N= 30 subjects, age 44 (8.4) y, BMI 28.5 (4.1) kg/m^2^ | Longitudinal, 3 months | Technology: segmental  Frequency: multifrequency  Device: BC-418, Tanita | Raw measures:  N/A  Estimates:  FM | Predictive equations for estimating body composition were not reported |
| Klement et al. | 2020a | N= 29 oncologic subjects age 29-78 y, BMI 19.9 – 45.2 kg/m^2^ | Longitudinal,  >2 months | Technology: segmental  Frequency: multifrequency  Device: mBCA 515/514, SECA | Raw measures:  PhA at 50 kHz  Estimates:  FM, FFM, TBW, ECW, and ICW | Predictive equations for estimating body composition were not reported |
| Klement et al. | 2020b | N= 8 oncologic subjects age 28-74 y, BMI 20.7 – 32.3 kg/m^2^ | Longitudinal,  >2 months | Technology: segmental  Frequency: multifrequency  Device: mBCA 515/514, SECA | Raw measures:  PhA at 50 kHz  Estimates:  FM, FFM, TBW, ECW, and ICW | Predictive equations for estimating body composition were not reported |
| Lodi et al. | 2020 | N= 8 females, age 25-35 y, BMI 27 (1.9) kg/m^2^ | Longitudinal,  10 days | Technology: foot-to-hand  Frequency: 50 kHz  Device: BIA-101, Akern | Raw measures:  N/A  Estimates:  FM | Predictive equations for estimating body composition were not reported |
| Buscemi et. | 2021 | N= 25 females and 12 males, mean age 43 (11) y, mean BMI 39.4 (6.3) kg/m^2^ | Longitudinal, 10-12 weeks | Technology: foot-to-hand  Frequency: 50 kHz  Device: BIA-101, Akern | Raw measures:  PhA  Estimates:  FM and FFM | Predictive equations for estimating body composition were not reported |
| Cincione et al. | 2021 | N= 17 females, age 28.5 (5.4) y BMI 31.2 (5.9) kg/m^2^ | Longitudinal, 45 days | Technology: segmental  Frequency: multifrequency  Device: TBF-300A, Tanita Co. | Raw measures:  N/A  Estimates:  FM, FFM, TBW, and SMM | Predictive equations for estimating body composition were not reported |
| Foppiani et al. | 2021 | N= 5 males and 1 female, mean age 54 y, mean BMI 22.9 (3.3) kg/m^2^ | Longitudinal, 30 days | Technology: segmental  Frequency: multifrequency  Device: Inbody 720, Biospace Co. | Raw measures:  N/A  Estimates:  FM, FFM, TBW, ECW, and ICW | Predictive equations for estimating body composition were not reported |
| Kämmerer et al. | 2021 | N= 29 females, age 38-64 y, BMI 18.1-35.4 kg/m^2^ | Longitudinal, 14 months | Technology: foot-to-hand  Frequency: multifrequency  Device: Nutriguard-M, Data Input GmbH | Raw measures:  PhA at 50 kHz  Estimates:  FM and BCM | Predictive equations for estimating body composition were not reported |
| Klement et al. | 2021 | N= 12 males and 6 females, age 38-77, BMI 20.2 – 35 kg/m^2^ | Longitudinal, < 2 months | Technology: segmental  Frequency: multifrequency  Device: mBCA 515/514, SECA | Raw measures:  PhA at 50 kHz  Estimates:  FM, FFM, TBW, ECW, and ICW | Predictive equations for estimating body composition were not reported |
| Li et al. | 2021 | N= 8 subjects, age 31.1 (3.6) y, BMI 29.8 (2.4) kg/m^2^ | Longitudinal, 12 weeks | Technology: segmental  Frequency: multifrequency  Device: Inbody 770, Biospace Co. | Raw measures:  N/A  Estimates:  FM and VFA | Predictive equations for estimating body composition were not reported |
| Paoli et al. | 2021a | N= 9 males, age 26.2 (5.1) y, BMI 27 (1.9) kg/m^2^ | Longitudinal,  2 months | Technology: foot-to-hand  Frequency: 50 kHz  Device: BIA-101, Akern | Raw measures:  N/A  Estimates:  FM and FFM | Predictive equations for estimating body composition were not reported |
| Paoli et al. | 2021b | N= 8 males, age 25.5 (2.5) y, BMI 24.7 kg/m^2^ | Longitudinal,  30 days | Technology: foot-to-hand  Frequency: 50 kHz  Device: BIA-101, Akern | Raw measures:  N/A  Estimates:  TBW, ECW, and ICW | Predictive equations for estimating body composition were not reported |
| Schiavo et al. | 2021 | N= 12 males and 12 females, mean age 39 (8.8) y, BMI 37.8 (4.9) kg/m^2^ | Longitudinal,  4 months | Technology: segmental  Frequency: multifrequency  Device: Jawon IOI 535, Cosmed. | Raw measures:  PhA at 50 kHz  Estimates:  FM, FFM, and TBW | Predictive equations for estimating body composition were not reported |
| Vidić et al. | 2021 | N= 20 males, age 42.7 (1.5) y, BMI 27 kg/m^2^ | Longitudinal,  8 weeks | Technology: segmental  Frequency: multifrequency  Device: Inbody 770, Biospace Co. | Raw measures:  N/A  Estimates:  FM and LBM | Predictive equations for estimating body composition were not reported |
| Al Aamri et al. | 2022 | N= 100 subjects (32% males and 68% females), age 38.4 (10.4) y, BMI 45.9 (8.6) kg/m^2^ | Longitudinal,  6 months | Technology: segmental  Frequency: multifrequency  Device: mBCA 515/514, SECA | Raw measures:  N/A  Estimates:  FM, SMM, and VFA | Predictive equations for estimating body composition were not reported |
| Barrea et al. | 2022a | N= 247 females, age 35.4 (10.5), BMI 37.3 (4.5) kg/m^2^ | Longitudinal,  45 days | Technology: foot-to-hand  Frequency: 50 kHz  Device: BIA-101, Akern | Raw measures:  R and Xc  Estimates:  FM and FFM | Predictive equations for estimating body composition were not reported |
| Barrea et al. | 2022b | N= 260 females, age 18-69 y, BMI 25.0-50.9 kg/m^2^ | Longitudinal,  30 days | Technology: foot-to-hand  Frequency: 50 kHz  Device: BIA-101, Akern | Raw measures:  R, Xc, and PhA  Estimates:  N/A |  |
| Camajani et al. | 2022a | N= 1 female, age 55 y, BMI 36.7 kg/m^2^ | Longitudinal,  6 weeks | Technology: foot-to-hand  Frequency: 5, 10, 50, 100, and 250 kHz  Device: Human Im Touch, DS Medica S.r.l. | Raw measures:  N/A  Estimates:  FM and FFM | Predictive equations for estimating body composition were not reported |
| Camajani et al. | 2022b | N=3 males and 21 females, age 56.3 (5.3) y, BMI 33.9 (3.4) kg/m^2^ | Longitudinal,  6 weeks | Technology: foot-to-hand  Frequency: 5, 10, 50, 100, and 250 kHz  Device: Human Im Touch, DS Medica S.r.l. | Raw measures:  N/A  Estimates:  FM, FFM, and BCM | Predictive equations for estimating body composition were not reported |
| Di Rosa et al. | 2022 | N= 135 subjects (34 males and 101 females), age 45.5 (11.6) y, BMI 33.5 (5.1) kg/m^2^ | Longitudinal,  3 months | Technology: foot-to-hand  Frequency: 50 kHz  Device: BIA-101, Akern | Raw measures:  PhA  Estimates:  FM, FFM, TBW, ECW, ICW, and BCM | Predictive equations for estimating body composition were not reported |
| Cincione et al. | 2022 | N= 40 subjects (20 males and 20 females), age 52.1 (1.7) y, BMI 33.4 (9.9) kg/m^2^ | Longitudinal, 30 days | Technology: segmental  Frequency: multifrequency  Device: Inbody 770, Biospace Co. | Raw measures:  N/A  Estimates:  FM, FFM, and TBW | Predictive equations for estimating body composition were not reported |
| Klement and Sweeney | 2022 | N= 7 oncologic subjects (5 males and 2 females), age 61-75 y, BMI 19.3-26.1 kg/m^2^ | Longitudinal, 42 months | Technology: segmental  Frequency: multifrequency  Device: mBCA 515/514, SECA | Raw measures:  PhA at 50 kHz  Estimates:  FM, FFM, TBW, ECW, ICW, and SMM | Predictive equations for estimating body composition were not reported |
| Mancin et al. | 2022 | N= 16 male soccer players, age 22.5 (2.) y | Longitudinal,  30 days | Technology: foot-to-hand  Frequency: 50 kHz  Device: BIA-101, Akern | Raw measures:  N/A  Estimates:  TBW and ECW | Predictive equations for estimating body composition were not reported |
| Sørlie et al. | 2022 | N= 9 females with lipedema, age 18–75 y, BMI: 30–45 kg/m^2^ | Longitudinal,  7 weeks | Technology: segmental  Frequency: multifrequency  Device: Inbody 720, Biospace Co. | Raw measures:  N/A  Estimates:  FM, FFM, TBW, and SMM | Predictive equations for estimating body composition were not reported |
| Valente et al. | 2022 | N= 23 migraine (1 male and 22 females), age 47.2 (15.1) y, BMI 26.9 (6.2) kg/m^2^ | Longitudinal,  3 months | Technology: foot-to-hand  Frequency: 50 kHz  Device: BIA-101, Akern | Raw measures:  PhA  Estimates:  FM, LM, TBW, ECW, and ICW | Predictive equations for estimating body composition were not reported |
| Valinejad et al. | 2022 | N= 36 males, age 20.7 (1.4) y, BMI 31.1 (3.9) kg/m^2^ | Longitudinal,  6 weeks | Technology: segmental  Frequency: multifrequency  Device: Inbody 3, Biospace Co. | Raw measures:  N/A  Estimates:  FM, and LBM | Predictive equations for estimating body composition were not reported |
| Verde et al. | 2022 | N =318 females, age 38.8 (14.4) y, BMI 35.8 (5.2) kg/m^2^ | Longitudinal,  45 days | Technology: foot-to-hand  Frequency: 50 kHz  Device: BIA-101, Akern | Raw measures:  R, Xc, and PhA  Estimates:  FM and FFM | Predictive equations for estimating body composition were not reported |
| Yang et al. | 2022 | N= 55 females with polycystic ovary syndrome, age 28.0 (5.0) y, BMI 31.0 (5.0) kg/m^2^ | Longitudinal,  12 weeks | Technology: segmental  Frequency: multifrequency  Device: Inbody 770, Biospace Co. | Raw measures:  N/A  Estimates:  FM and VFA | Predictive equations for estimating body composition were not reported |
| Barrea et al. | 2023a | N= 137 females, age 46.5 (10.1) y, BMI 37.0 (4.5) kg/m^2^ | Longitudinal,  45 days | Technology: foot-to-hand  Frequency: 50 kHz  Device: BIA-101, Akern | Raw measures:  PhA  Estimates:  FM, TBW, and ECW | Predictive equations for estimating body composition were not reported |
| Barrea et al. | 2023b | N= 324 females, age 39.3 (15.1) y, BMI 32.3 (4.2) kg/m^2^ | Longitudinal,  31 days | Technology: foot-to-hand  Frequency: 50 kHz  Device: BIA-101, Akern | Raw measures:  R, Xc, and PhA  Estimates:  FM, and FFM | Predictive equations for estimating body composition were not reported |
| Cincione et al. | 2023 | N= 73 subjects, age 33.4 (5.7) y, BMI 33.4 (5.7) kg/m^2^ | Longitudinal,  45 days | Technology: segmental  Frequency: multifrequency  Device: Tanita TBF-300A, Tanita Co. | Raw measures:  N/A  Estimates:  FM, FFM, and TBW | Predictive equations for estimating body composition were not reported |
| De Nucci et al. | 2023 | N= 58 females and 29 males, age 42.4 (12.9) y, BMI 35.6 (6.3) kg/m^2^ | Longitudinal,  8 weeks | Technology: foot-to-hand  Frequency: 50 kHz  Device: BIA-101, Akern | Raw measures:  N/A  Estimates:  FM, and FFM | Predictive equations for estimating body composition were not reported |
| Elsahoryi et al. | 2023 | N= 20 females, age 34.7 (3.3) y, BMI 33.2 (5.2) kg/m^2^ | Longitudinal,  8 weeks | Technology: segmental  Frequency: multifrequency  Device: Tanita TBF-300A, Tanita Co. | Raw measures:  N/A  Estimates:  FM, FFM, VFA, TBW, SMM, and MM | Predictive equations for estimating body composition were not reported |
| Kysel et al. | 2023 | N= 13 males, age 23.0 (5.0) y, BMI 26.1 (3.7) kg/m^2^ | Longitudinal,  8 weeks | Technology: segmental  Frequency: multifrequency  Device: Inbody 320, Biospace Co. | Raw measures:  N/A  Estimates:  FM, TBW, and MM | Predictive equations for estimating body composition were not reported |
| Merlino et al. | 2023 | N= 70 (58 females and 12 males) migraine subjects, age 46.1 (14.4), BMI 26.1 (5.7) kg/m^2^ | Longitudinal,  3 months | Technology: foot-to-hand  Frequency: 50 kHz  Device: BIA-101, Akern | Raw measures:  N/A  Estimates:  FM and FFM | Predictive equations for estimating body composition were not reported |
| Pandurevic et al. | 2023 | N= 16 polycystic ovary  syndrome females, age 28.1 (5.8) y, BMI 33.9 (3.8) kg/m^2^ | Longitudinal,  16 weeks | Technology: foot-to-hand  Frequency: 50 kHz  Device: BIA-101, Akern | Raw measures:  N/A  Estimates:  FM and FFM | FM and FFM were estimated using the equation proposed by Sun et al. (2003), as follow:   1. FFM (kg) = −10.68 + 0.65 × (H^2^/R) + 0.26 × Wt + 0.02 × R 2. FM = (Body mass − FFM) |
| Rinaldi et al. | 2023 | N= 29 females and 9 males, age 40.2 (14.9) y, BMI 33.8 (6.6) kg/m^2^ | Longitudinal,  16 weeks | Technology: foot-to-hand  Frequency: 50 kHz  Device: BIA-101, Akern | Raw measures:  N/A  Estimates:  FM and FFM | Predictive equations for estimating body composition were not reported |
| Rondanelli et al. | 2023 | N=1 female subject, age 22 y, BMI of 30.8 kg/m^2^ | Longitudinal,  4 months | Technology: N/A  Frequency: multyfrequency  Device: Zeus, Cosmed | Raw measures:  N/A  Estimates:  FM, FFM, and VFA | Predictive equations for estimating body composition were not reported |
| Tereshko et al. | 2023a | N =17 females and 4 males with migraine, age 44.1 (14.7) y, BMI 21.8 (2.7) kg/m^2^ | Longitudinal,  3 months | Technology: foot-to-hand  Frequency: 50 kHz  Device: BIA-101, Akern | Raw measures:  PhA  Estimates:  FM, FFM, ECW, and ICW | Predictive equations for estimating body composition were not reported |
| Tereshko et al. | 2023b | N =58 females and 12 males with migraine, age 45.9 (14.8) y, BMI 26.7 (5.9) kg/m^2^ | Longitudinal,  3 months | Technology: foot-to-hand  Frequency: 50 kHz  Device: BIA-101, Akern | Raw measures:  N/A  Estimates:  FM and FFM | Predictive equations for estimating body composition were not reported |
| Verde et al. | 2023a | N =248 females, age 38.8 (14.1) y, BMI 36.0 (5.2) kg/m^2^ | Longitudinal,  31 days | Technology: foot-to-hand  Frequency: 50 kHz  Device: BIA-101, Akern | Raw measures:  R, Xc, and PhA  Estimates:  FM and FFM | Predictive equations for estimating body composition were not reported |
| Vetrani et al. | 2023 | N= 86 females, age 36.8 (14.1) y, BMI 35.9 (5.2) kg/m^2^ | Longitudinal,  45 days | Technology: foot-to-hand  Frequency: 50 kHz  Device: BIA-101, Akern | Raw measures:  R and Xc  Estimates:  FM and MM | Predictive equations for estimating body composition were not reported |
| Vinciguerra et al. | 2023 | N= 18 females and 8 males, age 50.3 (10.3) y, BMI 37.5 (5.5) kg/m^2^ | Longitudinal,  19 weeks | Technology: segmental  Frequency: multifrequency  Device: MC-180-MA, Tanita Co. | Raw measures:  N/A  Estimates:  FM, FFM, TBW, ECW, and ICW | Predictive equations for estimating body composition were not reported |
| Note: BIA, bioelectrical impedance analysis; BMI, body mass index; R, resistance; Xc, reactance, Z, impedance; PhA, phase angle; FM, fat mass; FFM, fat-free mass; LM, lean mass; TBW, total body water; LBM, lean body mass; VFA, visceral fat area; ECW, extracellular water; ICW, intracellular water; SMM, skeletal muscle mass; MM, muscle mass; ALSM, appendicular lean soft mass; BCM, body cell mass. | | | | | | |
